# Ultrasensitive Determination of Glial-Fibrillary-Acidic-Protein (GFAP) in Human Serum-Matrix with a Label-Free Impedimetric Immunosensor

**DOI:** 10.3390/bios12121165

**Published:** 2022-12-14

**Authors:** Goksu Ozcelikay, Fariba Mollarasouli, Mehmet Altay Unal, Kıvılcım Gucuyener, Sibel A. Ozkan

**Affiliations:** 1 Department of Analytical Chemistry, Ankara University, Ankara 06560, Turkey; 2 Department of Chemistry, Yasouj University, Yasouj 75918-74831, Iran; 3 Stem Cell Institute, Ankara University, Ankara 06520, Turkey; 4 Department of Pediatric Neurology, Gazi University, Ankara 06510, Turkey

**Keywords:** label-free impedance immunosensor, screen printed carbon electrode (SPCE), Au nanoparticles (Au NPs), glial fibrillary acidic protein (GFAP)

## Abstract

In this work, immobilizing anti-GFAP antibodies via covalent attachment onto L-cysteine/gold nanoparticles that were modified with screen-printed carbon electrodes (Anti-GFAP/L-cys/AuNps/SPCE) resulted in the development of a sensitive label-free impedance immunosensor for the detection of Glial Fibrillary Acidic Protein (GFAP). The immunosensor’s stepwise construction was studied using cyclic voltammetry (CV) and electrochemical impedance spectroscopy (EIS). L-cysteine was chosen as the linker between GFAP antibodies and Au NPs/SPCE because it enables the guided and stable immobilization of GFAP antibodies, thus resulting in increased immunosensor sensitivity. As a redox probe, 5 mM of [Fe(CN)6]3−/4− was used to measure the electron–transfer resistance (Ret), which was raised by the binding of antigens to the immobilized anti-GFAP on the surface of the modified electrode. A linear correlation between Rct and GFAP concentration was achieved under optimum conditions in the range of 1.0–1000.0 pg/mL, with an extraordinarily low detection limit of 51.0 fg/mL. The suggested immunosensor was successfully used to detect the presence of GFAP in human blood serum samples, yielding good findings. As a result, the proposed platform may be utilized to monitor central nervous system injuries.

## 1. Introduction

Detecting brain-specific proteins in peripheral blood or cerebrospinal fluid (CSF) may assist in estimating the level of brain damage and the prognosis of the condition. A variety of central nervous system (CNS) cell types, including astrocytes and ependymal cells, produce a glial-fibrillary-acidic-protein (GFAP), which is a type III intermediate filament (IF) protein, with a molecular mass between 40 and 53 kDa [1]. It was discovered that GFAP might be found in the blood after brain damage. This protein is normally not released and circulated in response to cell death or damage. It may be a biomarker for traumatic brain injury (TBI) since it is released when the CNS is damaged. GFAP levels in trauma patients are then monitored for 21 days. GFAP releases are associated with increased intracranial pressure (ICP), and the extent of brain damage detected by CT [2]; therefore, we may ask, generally, what is the GFAP level in blood?

GFAP levels were significantly higher in samples from AD patients and in AD pool samples than in samples from the healthy individuals pool; this is in accordance with what is reported in the literature [3]. Serum GFAP levels were significantly higher in patients with epileptic seizures compared with healthy controls. A cut-off point of 2.71 ng mL^−1^ was found to help optimally differentiate between epileptic seizures and psychogenic non-epileptic seizures [4]. GFAP found in the serum of patients with mild traumatic brain injuries and concussions is elevated to a concentration of 30−100 pg mL^−1^, as opposed to <1−10 pg mL^−1^ in healthy subjects. This concentration also reaches 1.5−5 ng mL^−1^ for patients with moderate traumatic brain injuries [5].

Furthermore, GFAP has a good diagnostic ability to predict post-injury outcomes. As a result, there is a critical demand for quick and low-cost assays that may be utilized immediately from a patient’s sample to monitor GFAP levels at the point of care. The ELISA [6], electrochemiluminescent (ECL) [7], dissociation-enhanced lanthanide fluorescence immunoassay (DELFIA) system [8], time-resolved fluorescent lateral flow immunoassay (TRF-LFIA) [9], and chemiluminometric immunoassay [1] are a few current examples in recent works that have been published addressing GFAP measurements; however, the clinically relevant concentrations cannot be covered by their sensitivities and detection ranges with expensive equipment, which restricts the use of the abovementioned methods for an accurate diagnosis of GFAP. Numerous papers on nanoparticles have recently been published for GFAP protein measurement by electrochemical biosensors [5,10,11,12,13,14]. Electrochemical immunosensors offer various advantages over other techniques, including ease of use, simplicity of instrumentation, minimal sample amounts, high sensitivity, and selectivity.

Nanotechnologies and nanomaterials have received a lot of interest because of the inherent properties of the distinct nanoparticles that allow for their broad use in fields such as sensor development. Gold nanoparticles (Au NPs) are the most commonly employed nanomaterials due to their outstanding conductivity, exceptional catalytic capabilities, and enormous specific surface area [15]. Electron transport between analytes and the modified surface can be accelerated by Au NPs placed on the electrode surface. As a result of their excellent electron transfer kinetics, gold NPs with modified electrodes in various shapes and sizes have been widely used as a backing material for electrochemical analysis. The combination of L-cys and Au NPs is likely to exhibit appealing qualities due to the synergistic impact of L-cys and Au NPs, such as high effective surface area and stability compared with those without Au NPs [16]; however, to produce a biosensor with improved features, such as stability and selectivity, the sensing molecule’s attachment and interaction with the solid surface are critical [17,18]. Self-assembled monolayers (SAMs) enable the attachment of biomolecules to the surface of an electrode, and electrochemical detection techniques result in cheap costs, quick reaction times, enhanced sensitivity, and enhanced mobility [18,19,20]. These methods have recently been used to fabricate biosensors. Additionally, SAMs are simple to create, and their characteristics may be easily changed by switching their functional groups to ones that allow for the immobilization of biomolecules; however, to connect a biomolecule with a SAM, it generally has to be either activated or modified further, which might compromise the integrity of the SAM or biomolecule and cause denaturation and loss of function [18,21,22].

In this study, L-cysteine was electropolymerized on an AuNP’s modified screen printed electrode (Au NPs/SPCE) using a self-assembled approach that was based on a thiols group via CV technique, thus allowing for orientated antibody immobilization. L-cysteine was chosen as the linker between GFAP antibodies and Au NPs/SPCE because it allows for the direct and stable immobilization of GFAP antibodies. Moreover, its amino group can possibly connect with the antibody via a dehydration–condensation process [23]. This is useful in the electrochemical detection of GFAP without the use of labels. Label-free assessments of different biomarkers are desirable because they offer real-time monitoring. It is vital to keep in mind that creating label-free immunosensors has several benefits, including a significant reduction in detection time and cost savings by eliminating the labeling stage [24]. The EIS response must be amplified, and strategies for raising the transfer resistance must be developed to design a sensor without a label that can provide a strong electrochemical signal. In order to replace the GFAP assay in the clinical diagnosis of TBI, the proposed immunosensor system can be used.

## 2. Materials and Methods

### 2.1. Chemicals and Reagents

The screen-printed carbon electrode (SPCE) that was used as a working electrode was supplied by the Metrohm Dropsens (Oviedo, Spain). The carbon counter electrode and the Ag/AgCl reference electrode are integrated into the triple system of the electrode. The GFAP and anti-GFAP were supplied by Sigma-Aldrich (St. Louis, MO, USA). The GFAP and anti-GFAP proteins were prepared and aliquotted with phosphate buffer saline (PBS, pH 7.4) and stored at −20 °C. Gold nanoparticles (AuNPs), L-cysteine (l-cys), bovine serum albumin (BSA), and EDC/NHS (N-(3-Dimethylaminopropyl)-N-ethylcarbodiimide hydrochloride/N-hydroxysuccinimide solutions were supplied from Sigma-Aldrich (St. Louis, MO, USA). A 5 mM [Fe(CN)_6_] ^4−/3−^ redox probe was prepared in the presence of 0.1 M KCl in Milli-Q water. All analyses were performed at room temperature (25 °C).

### 2.2. Equipments

Electrochemical impedance spectroscopy (EIS) was utilized to characterize the GFAP/BSA/anti-GFAP/L-cys/AuNPs/SPCE immunosensor and to determine GFAP in the buffer and human serum samples. EIS measurements were performed in a frequency range of 0.1 Hz to 100 kHz, with a 25 mV amplitude using an Autolab Potentiostat/Galvanostat (Autolab Instruments, Herisau, Switzerland) with NOVA 2.1.2 software to assess charge transfer resistance values. The determination of GFAP was carried out by immersing the immunosensor in a 5 mmol L^−1^ Fe(CN)_6_^3−/4−^ solution (a mixture of 5 mM Fe(CN)_6_^4−^ (ferrocyanide) and 5 mM Fe(CN)_6_^3−^ (ferricyanide)) as a probe prepared in 0.1 mol L^−1^ KCl. The analytical signals were obtained from the change in the charge transfer resistance value before and after the antigen–antibody reaction (ΔRct). Between every measurement, the immunosensor was washed sequentially with Milli-Q water and with PBS buffer (pH 7.4).

### 2.3. Standard and Sample Preparation

#### 2.3.1. Preparation of Gold Nanoparticles Modified SPCE

Before beginning the advanced immobilization steps of the biosensor, the disposable SPCE was first activated in a 0.5 M H_2_SO_4_ solution at a scanning rate of 50 mV/s between −0.2 V and 1.2 V (cycle 20). The activated -OH groups on the surface were obtained and washed with ultrapure water. The 5µL AuNPs were dropped on the SPCE surface and left to dry at room temperature.

#### 2.3.2. Preparation of the Synthetic Human Serum Sample

The GFAP-spiked serum sample was made up of 5.4 mL acetonitrile, 3.6 mL plasma, and 1.0 mL GFAP stock solution at 10 ng/mL. First, the mixed solution was centrifuged for 25 min at 3000 rpm to precipitate the protein residues in the solution. Then, the supernatants were removed before measurement.

#### 2.3.3. Design of the Immunosensor

**Scheme 1:**
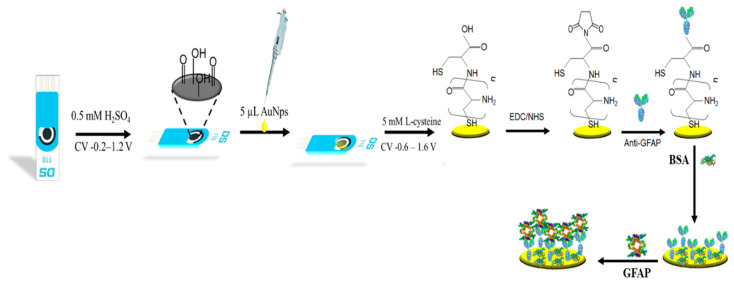
The illustration of the developed GFAP immunosensor.

The electropolymerization of L-cysteine (L-cys) and the immobilization steps of anti-GFAP on the L-cys modified AuNPs/SPCE are represented in Scheme 1. First, the AuNPs/SPCE were electropolymerized in a 5 mM L-cys solution. The electropolymerization process occurred in the range of −0.6 V–1.6 V (100 mV/s, 20 cycles) with the CV [25]. After this treatment, the electrode surfaces were immersed in Milli-Q water and a PBS solution with a pH of 7.4 to remove unbound amino thiols. The carboxyl ends of the L-cys were activated and they easily interacted with the –NH_2_ groups of the antibody as a result of the EDC/NHS (4:1 (*v/v*)) solution.

Then, the L-cys/AuNPs/SPCE surface was exposed to the GFAP antibody solution, and the immobilization process was maintained in each step. Moreover, 1% BSA was used to block the free –COOH groups and the non-interacting –NH_2_ groups of the GFAP antibody [26]. After each step, the anti-GFAP/L-cys/AuNPs/SPCE surfaces were gently washed with Milli-Q water and PBS. Finally, the prepared biosensors were kept in a refrigerator (4 °C) until the GFAP biomarker was measured. The surfaces of the bare SPCE and the modified electrodes were referred to as SPCE and SPCE/AuNPs, SPCE/AuNPs/L-cys, SPCE/AuNPs/L-cys/, SPCE/AuNPs/L-cys/anti-GFAP/BSA, respectively (Scheme 1).

## 3. Results

### 3.1. Electrochemical Characterization of the Immunosensor

EIS is performed to evaluate the fluctuations in interface properties between the electrolyte and electrode surface by means of a redox probe, Fe(CN)_6_^3−/4−^. EIS techniques are generally used to brighten the enzyme–substrate, antibody–antigen interactions, or DNA hybridizations.

**Figure 1:**
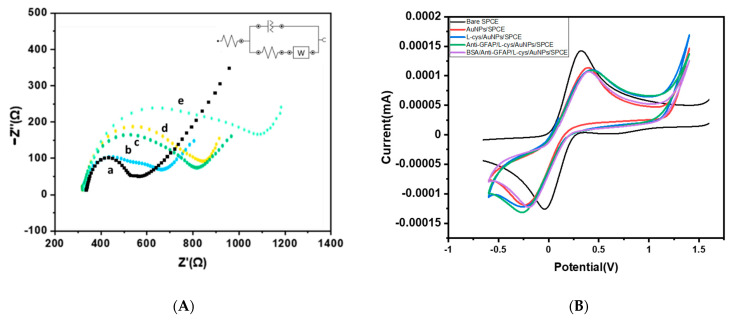
The Nyquist diagrams (**A**) showing GFAP immunosensors, step-by-step (a: Bare SPCE; b: AuNPs/SPCE; c: L-cys/AuNPs/SPCE;d: Anti-GFAP/L-cys/AuNPs/SPCE; e: BSA/Anti-GFAP/L-cys/AuNPs/SPCE) and cyclic voltammograms (**B**).

The EIS curves in Figure 1A show that the semi-circular diameter, indicated as R_ct_, of AuNps/SPCE (299.81 Ω) in the Nyquist diagram was quite low because of its highly conductive properties. Moreover, the R_ct_ of L-cys/AuNps/SPCE (468.32 Ω) was higher than AuNps/SPCE. After electropolymerization, the -COOH groups of L-cys were activated with EDC/NHS (4:1 (*v/v*)) solution. Then, the anti-GFAP solution was immobilized on the polymerized L-cys surface and covalent interactions between the active carboxyl and amine groups occurred; therefore, the R_ct_ of the Anti-GFAP/L-cys/AuNps/SPCE increased to 494.2 Ω. As the covalent immobilization of anti-GFAP generated a barrier effect on the surface, the redox probe had difficulty diffusing the surface as a result of this layer. Finally, a BSA blocking agent was used to block active carboxyl groups which could not bind to the antibody. An increase in charge transfer resistance from 494.2 Ω to 654.43 Ω was observed due to the fact that the electrode surface provided a higher degree of insulation. The EIS results are in accordance with those provided by cyclic voltammograms (Figure 1B).

### 3.2. Optimization of the Experimental Variables Involved in the Preparation of the Immunosensor

All parameters, such as the AuNPs’ modifier amount, the concentration of anti-GFAP, anti-GFAP immobilization time, the BSA concentration, BSA immobilization time, and GFAP immobilization time, were evaluated in order to construct a linear and sensitive GFAP immunosensor. The largest S/B ratio between the charge transfer resistance differences (ΔRct), which was measured with the immunosensor in the absence of (B) and in the presence of GFAP standard solutions containing 100 pg mL−1 (S) was selected testing variables such as the concentration of anti-GFAP, anti-GFAP immobilization time, BSA concentration, and BSA immobilization time. 

**Figure 2:**
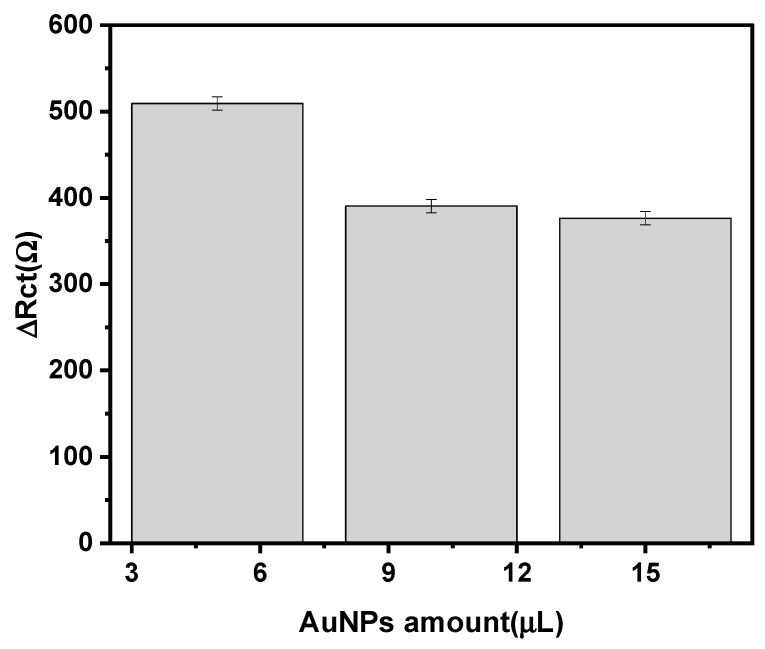
The optimization of the AuNPs’ modifier amount.

The ability of AuNPs to permit fast and direct electron transfer has been proven to increase the surface area of the electrode. This use of AuNPs is known as mediator-free electrochemical sensing. According to Figure 2, 5 µL was obtained for the best response. First, a AuNPs solution was dropped onto an electrochemically cleaned SPCE, which was allowed to dry in ambient air, and rinsed with distilled water and PBS to obtain the AuNPs’ modified SPCE (AuNPs/SPCE). The 5 µL drop volume of the AuNPs solution was enough to cover the 4 mm diameter of the working electrode. Second, the AuNPs/SPCE was performed via CV scanning from −0.6 V to 1.6 V (scan rate of 0.25 V s^−1^, 20 cycles) in 5 mM L-cys to obtain L-cys/AuNPs/SPCE. Following that, the L-cys, AuNPs, and SPCE were immersed in Milli-Q water and a PBS solution.

**Figure 3:**
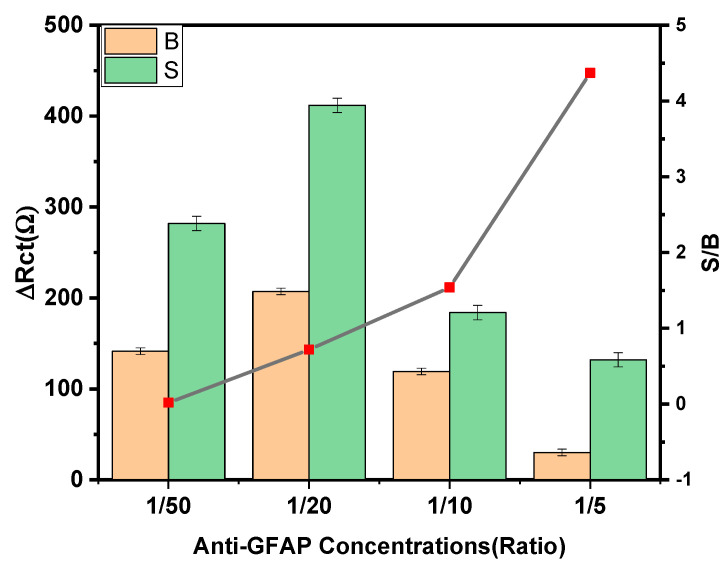
Effect of anti-GFAP loading with the antibody solution on R_ct_ responses measured with the Anti-GFAP/L-cys/AuNPs/SPCE for 0 (B) and 100 (S) pg mL^−1^ GFAP standard solutions.

The concentration of the GFAP antibody is another parameter that needs to be optimized. Four different anti-GFAP concentrations (1/50, 1/20, 1/10,1/5) were optimized to demonstrate the influence of anti-GFAP on the signal of the L-cys/AuNPs/SPCE immunosensor using EIS results. As can be observed in Figure 3, the S/B ratio notably increased with the anti-GFAP loading, up to 1/5, because non-specific signals decreased when loading a 1/5 ratio of anti-GFAP. The best value was observed when 1/5 of an anti-GFAP concentration was utilized (Figure 3).

**Figure 4:**
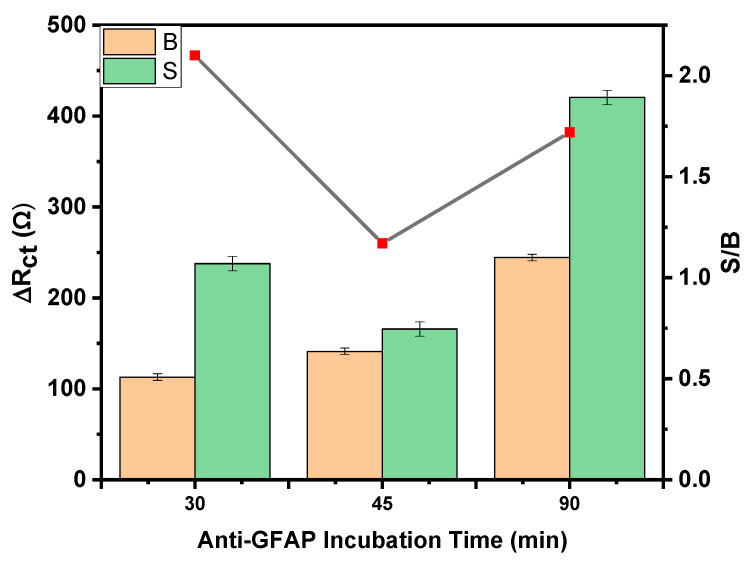
Effect of the anti-GFAP incubation time with the antibody solution on the R_ct_ responses. Measured with the Anti-GFAP/L-cys/AuNPs/SPCE for 0 (B) and 100 (S) pg mL^−1^ GFAP standard.

After the anti-GFAP antibody concentration was optimized, the optimum antibody incubation time was investigated. The short incubation time was preferable due to nonspecific adsorptions. The 30 min incubation period was adequate for the anti-GFAP immobilization to L-cys/AuNPs/SPCE and antibody–antigen interactions (Figure 4).

**Figure 5:**
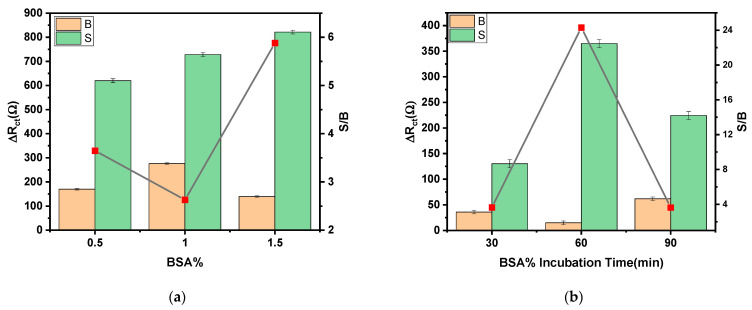
Effect of BSA concentration (**a**) and incubation time with BSA (**b**) on the R_ct_ responses measured with the Anti-GFAP/L-cys/AuNPs/SPCE for 0 (B) and 100 (S) pg mL^−1^ GFAP standard solutions.

**Figure 6:**
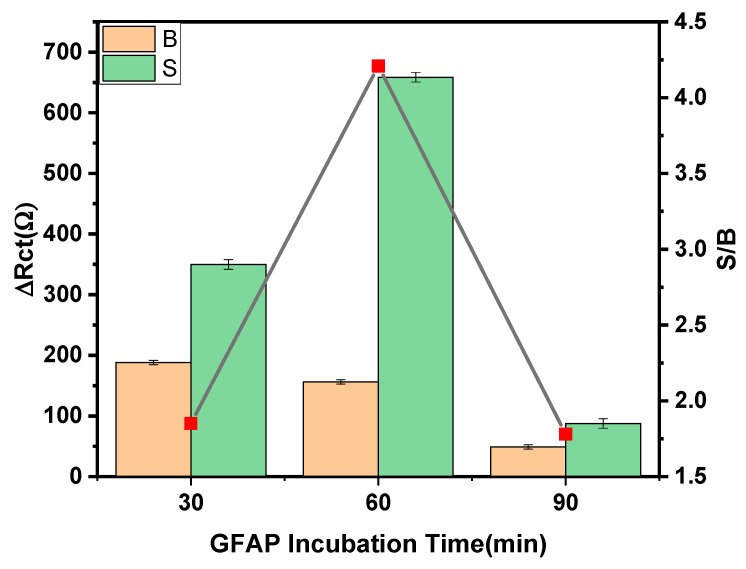
Optimization study of GFAP incubation time.

In order to block the free and reactive glycydoxy ends to minimize non-specific adsorptions, the biosensor was rinsed into a solution of BSA. As a result, the optimum BSA concentration was found to be 1.5% (Figure 5a). Moreover, the highest value of ∆Rct was obtained when BSA was incubated on the Anti-GFAP/L-cys/AuNps/SPCE surface for 60 min. Hence, the abovementioned values were chosen as the optimum values. Regarding the effect of the incubation time (Figure 6), the S/B values notably increased up to 60 min, and they decreased sharply over longer periods of time. The 60 min was adequate to allow for antibody and antigen interactions (Figure 6). The obtained results for these optimization studies are summarized in Table 1.

### 3.3. Analytical Performances for the Designed GFAP Biosensor

**Figure 7:**
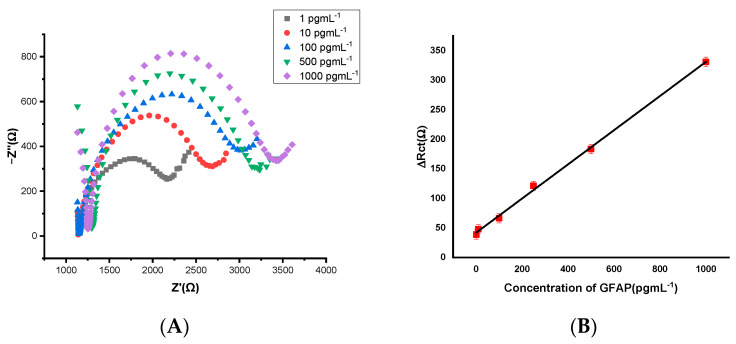
The analytical performance of the GFAP biosensor with the EIS results (**A**) and calibration curve, (**B**) type of signal transducer, application of nanomaterials, detection technique, type of bioreceptor, type of redox marker.

To achieve the calibration plot, the different concentrations of the GFAP solution were incubated on the BSA/Anti-GFAP/L-cys/AuNPs/SPCE surface, and ΔRct values were recorded using the EIS technique. Under the optimized experimental conditions, the plot of the R_ct_ differences in the absence of, and in the presence of, the GFAP standard solution (ΔRct), versus the GFAP concentrations, exhibited linear (R^2^ = 0.998) behaviors between 1 pg mL^−1^ and 1000 pg mL^−1^, with slope and intercept values of (0.289) Ω/pg mL^−1^ and (41.68) Ω, respectively (Figure 7). According to the 3s_b_ and 10s_b_ criteria, where s_b_ was estimated as the standard deviation (n = 3) for measurements in the absence of GFAP, the limit of detection (LOD) and the limit of quantification were obtained as 0.051 pg mL^−1^ and 0.168 pg mL^−1^, respectively. As seen in Table 2, according to analytical performances, the developed GFAP immunosensor has a high level of sensitivity.

ΔRct(Ω) = 0.289 ± 0.0058 [GFAP(pgmL^−1^)] + 41.68 ± 2.724 R^2^ = 0.998 (1)

### 3.4. Application of the GFAP Immunosensor in a Synthetic Human Serum Sample

**Figure 8:**
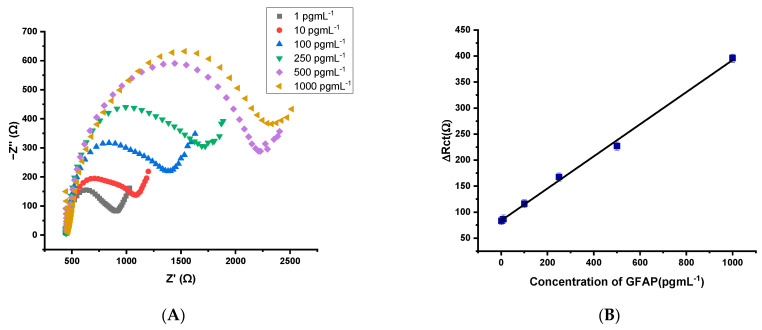
Application of the developed GFAP biosensors with the EIS results, (**A**) and the calibration curve (**B**) in the synthetic serum sample.

The developed Anti-GFAP/L-cys/AuNPs/SPCE biosensor was used for detecting the GFAP concentration in a synthetic human male AB plasma sample. The diluted concentrations were prepared from the synthetic serum sample for the calibration curve of GFAP, as shown in Figure 8.

Under the optimized experimental conditions, the plot of the R_ct_ differences in the absence of, and in the presence of, the GFAP standard solution (ΔRct) versus the GFAP concentrations exhibited a linear (R^2^ = 0.997) behavior between 1 pg mL^−1^ and 1000 pg mL^−1^, with slope and intercept values of (0.308) Ω/pg mL^−1^ and (83.74) Ω, respectively. The limit of detection (LOD) and quantification (LOQ) were obtained as 0.2 pg mL^−1^ and 0.66 pg mL^−1^, respectively.

ΔRct(Ω) = 0.308 ± 0.0077 [GFAP(pgmL^−1^)] + 83.74 ± 3.647 R^2^ = 0.997 (2)

### 3.5. Selectivity Performance of the Immunosensor

**Figure 9:**
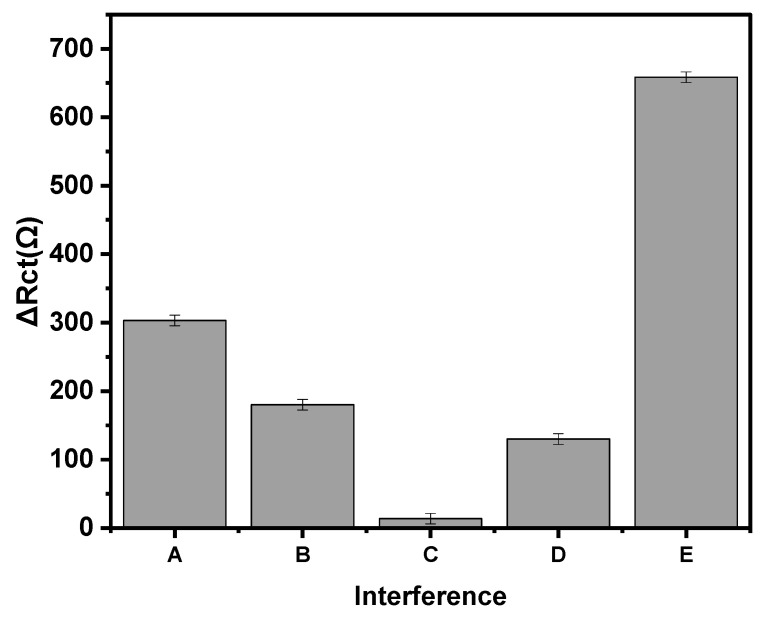
Selectivity of the GFAP biosensor in the presence of different biomolecules in the serum sample. ΔR_ct_ detected after immobilization of 10 ngmL^−1^ CRP (A), 2 mM Cholesterol (B), 2 mM Lysine (C), 2 mgmL^−1^ Hemoglobin (D), and 100 pgmL^−1^ GFAP (E) prepared in PBS in the presence of [Fe(CN)_6_]^3−/4−^ with EIS.

The selectivity study was performed to understand whether there are non-specific interactions or interferences. For this purpose, under optimum conditions, the BSA/Anti-GFAP/L-cys/AuNPs/SPCE was treated with standard solutions of 0.0, and 100 pg mL^−1^ of GFAP, that were prepared in the absence of, and in the presence of, each non-target protein, such as Lysine (2 mM), CRP (10 ngmL^−1^), Hemoglobin (2 mgmL^−1^), and Cholesterol (2 mM). The obtained ΔRct values from the biosensor responses were discussed and compared with each other. Figure 9 confirm that the proposed immunosensor represents a significantly higher specificity response to GFAP (100 pg mL^−1^) compared with other counterparts (Lysine, CRP, Hemoglobin, Cholesterol) in relatively similar concentrations.

The selectivity studies were performed in previous works. Khetani et al. reported that the polyethyleneimine (PEI) coated graphene screen-printed electrode was developed for the determination of GFAP. To test the selectivity, the developed biosensor was applied in the presence of the different biochemicals (NDMA, GABA, glutamate, and S100β) found after CNS injury. The immunosensor performance was highly selective in similar tests that were performed with prepared samples [5]. Wang T. et al. developed the MIP-based electrochemical sensors for the determination of GFAP. The interfering agents (Lysozyme, hemoglobin (Hb), insulin, and bovine serum albumin (BSA)) were applied for an investigation of selectivity. It was reported that compared with other proteins, an extremely sensitive response to GFAP was observed for the MIP/SPCE, thus indicating that the GFAP was specifically integrated with the complementary sites in the imprinted polymer [28]; therefore, their results are also supported by the obtained results.

The storage stability of the BSA/Anti-GFAP/L-cys/AuNPs/SPCE was controlled with 5 mM [Fe(CN)_6_]^3−/4−^. No significant differences between the measured S/B ratio values were found for the impedimetric signals recorded for 0 and 100 pg mL^−1^ GFAP for at least 15 days.

## 4. Conclusions

We constructed a L-cys functionalized AuNPs-based SPCE immunosensor for extremely sensitive GFAP detection. There are several advantages to the use of L-cys on AuNPs-based SPCE. L-cys can be easily electro-polymerized on the AuNPs/SPCE surface with greater uniformity, thus resulting in better control over sensor accuracy and precision. The L-cys surface functional group serves to immobilize anti-GFAP antibodies on the immunosensor. In this study, L-cys was explored as a substrate for the immunization of GFAP antibodies. Moreover, gold nanoparticles can function as electron donors to enhance electron transfer and increase conductivity. This strategy exhibits an attractive analytical performance with a LOD of 51.0 fgmL^−1^. As emphasized in Table 2, the herein-suggested approach has delivered a lower limit of detection and a larger range of GFAP analysis than most similar research that has been recently published. The performance of the suggested sensor, when compared in terms of its critical sensor characteristics with previously reported GFAP sensors, clearly demonstrated significant benefits. To test the selectivity, the developed biosensor was successfully applied in the presence of different biochemicals found after CNS injury. The obtained results were supported by previous works.

## Figures and Tables

**Table 1:** Experimental variables optimized in preparation for the Anti-GFAP/L-cys/AuNPs/SPCE immunosensor.

Parameters	Checked Range	Selected Condition
AuNp Modifier Amount	5–15 µL	5 µL
Anti-GFAP concentration	1–10 kDa	10 kDa
Anti-GFAP incubation time	30–90 min	30 min
BSA%	0.5–1.5%	1.5%
BSA% incubation time	30–90 min	60 min
GFAP incubation time	30–90 min	60 min

**Table 2:** The comparison of GFAP with previous studies.

Method	Signal	Detection	Bioreceptor	Redox Marker	LDR	LOD	Ref.
	Tranducer	Technique					
ELISA	-	Enzyme-linked immunosorbent assays	-	-	0–14 ng/mL	0.79 ng/mL	[27]
Enhanced lanthanide fluorescence immunoassay	-	DELFIA^®^ 1234 fluorometer	-	-	0.5–2 μg/mL	0.2 μg/mL	[8]
MIP/MWCNT/SPCEs	SPCE	DPV	MIP	[Fe(CN)_6_] ^−3/−4^	0.2–10 μg/mL	0.04 μg/mL	[28]
PEI/GO/SPCE	graphene screen-printed electrode	EIS	GFAP antibody	[Fe(CN)_6_] ^−3/−4^	1–100 pg/mL	N. M	[5]
Graphene@PEDOT:PSS/GIPEC electrode	Homemade three-PET electrode	EIS	GFAP antibody	[Fe(CN)_6_] ^−3/−4^	1 pg/mL–10 ng/mL	281.7 fg/mL	[11]
automated PMMA layer microfluidic platform	Homemade screen-printed electrode	EIS	GFAP anti-body	[Fe(CN)_6_] ^−3/−4^	10–1000 pg/mL	3 pg/mL	[12]
Magnetoimpedance biosensor	Microfluidic chips	Magnetoimpedance	GFAP antibody	PBS	0.01 .0 ng/mL.	0.01 ng/mL	[29]
Anti-GFAP/L-cys/AuNPs/SPCE	SPCE	EIS	GFAP anti-body	[Fe(CN)_6_] ^−3/−4^	1–1000 pg/mL	0.051 pg/mL	This work

GO: Graphene oxide; PEI: polyethylenimine; PEDOT:PSS: poly(3,4-ethylenedioxythiophene) polystyrene sulfonate; PMMA:polymethyl methacrylate; PET: polyethylene terephthalate; MWCNT: Multiwalled carbon nanotube; MIP: Molecularly imprinted polymer.

## Data Availability

The data are confidential.
